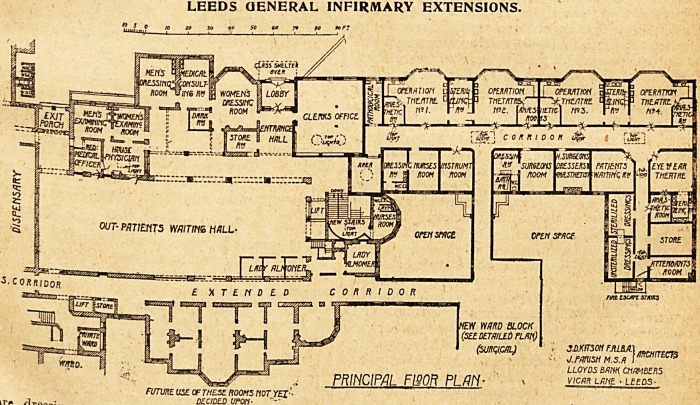# Leeds General Infirmary Extensions

**Published:** 1917-04-14

**Authors:** 


### HOSPITAL ARCHITECTURE AND CONSTRUCTION.

### LEEDS GENERAL INFIRMARY EXTENSIONS.

A glance at the block plan which we publish to-day will make clearer than any description the remarkable growth of this hospital. The original building comprised three ward pavilions on the north side of what is now a courtyard but what was intended by the architect, Sir Gilbert Scott, for a winter garden, and two ward pavilions on the south, and so satisfied was the then governing body of the sufficiency of the new building that they actually sold off some £15,000 worth of "surplus" land.

It will be seen by the plan that the site has been extended both to the west and to the north, so that it is considerably more than double the area of the original site.

The great increase in population of Leeds and the adjacent townships which rendered necessary the addition of a new out-patient department and an additional ward pavilion and isolation block in 1892, had in 1913 reached a figure nearly double that of 1868, when the hospital was opened for patients. The following figures will show the increase of work during the period in question.

In 1870, the first full year's work after the completion of the new building, 2,548 in-patients and 7,496 out-patients were treated.

In 1913 the corresponding figures were 9,019 in-patients and 40,227 out-patients.

To cope with this increased work it became necessary to acquire additional land, and the only possible way to do this was to expand in an easterly direction. Accordingly, some 21,552 yards of land and buildings were bought by the hospital authorities, part of which was resold to the Corporation in order that the new road of approach should be constructed.

LEEDS GENERAL INFIRMARY EXTENSIONS

The extension of the out-patient department is got by projecting a wing in an easterly direction from the north-east end of the existing building.

This wing is two storeys in height, the upper floor being occupied by the new suite of operation theatres.

The main entrance to the out-patient department is on the floor above the new extensions; the rooms on this floor on the north side of the hall are now used only for medical patients. The rooms at the east end of the hall have been cleared out and a large staircase constructed in the space thus obtained.

The staircase gives access to the new out-patient rooms, which comprise an ophthalmic department containing a large consulting-room, minor operation room, dark-room with thirteen compartments, and a small waiting-room for patients under treatment, surgeons' consulting-room with examination-rooms for patients of both sexes, aural department with operation-room and recovery-room used in common with the surgical department, a wide corridor to serve as waiting-room, with sanitary offices for each sex.

Alongside the staircase is a lift which connects the two floors.

The upper floor of this block contains, on the north

### LEEDS GENERAL INFIRMARY EXTENSIONS.

side, four operation theatres, each with its anæsthetic room, two sterilising-rooms to serve the four theatres, and a pathological room; on the south side of the corridor

### LEEDS GENERAL INFIRMARY EXTENSIONS.

are dressing-rooms for nurses, a surgeons' room, with dressing-room, bathroom, and w.c. adjoining, a dressing-room for house surgeons, anæsthetists, and dressers, a patients' waiting-room, and an instrument-room. Facing east is the operation theatre for eye and ear cases, with anæsthetic and sterilising room adjoining; and adjoining the last is a store and attendants' room. The rest of this wing is taken up by the sterilising-room for dressings. This room is divded into two, the steriliser being placed centrally in the division wall, so that unsterilised dressings are put into the apparatus in one side of the division wall and taken out on the other side.

The new ward pavilion is approached by an extension eastward of the corridor running east and west at the south side of the courtyard and the out-patient department. At the north side of the corridor is a staircase with

LEEDS GENERAL INFIRMARY EXTENSIONS—(*continued*).

a small store adjoining. Opposite this is a lift which opens into the main corridor. The ward offices include store for patients' clothes, private ward, ward kitchen, testing-room, bathroom, w.c. for nurses, two w.c.s for patients, and a sink-room. The latter three are arranged in a wing separated by a cross-ventilated lobby. The ward contains thirty-two beds, and at the south end is a wide balcony, off which an emergency staircase for escape in case of fire is arranged. The block is three storeys in height, all floors being alike.

The floors of the wards are laid with wax-polished teak boards. The walls have a pale cream-coloured tile dado 5 feet high, with Parian cement above; and the ceilings, which are kept free from projecting beams, are finished in the same material. The wards are heated by two pairs of central ventilating grates with descending flues, augmented by hot-water pipes. The private ward, which is intended to serve at times as sister's room, is finished in a similar way to the large ward. The other rooms have terrazzo floors and tiled dados, the tiling in the case of the sink-room and kitchen being carried up to the ceiling.

The roof over the ward block is flat and paved with asphalt, and is intended to be used for patients, to which end the lift is carried up to the roof level.

The extension of the nurses' home provides for fifty-two bedrooms for nurses, with a day-room for nurses and a bedroom and sitting-room for the matron.

The boiler-house block, at the extreme north corner of the site, and therefore on the highest ground, contains two 30 feet by 8 feet Lancashire boilers, engine-house, pump-room, mechanics' workshop, and small office, and is connected with the main buildings by a duct in which all pipes are run.

The whole of these very interesting and well-arranged extensions have been carried out from plans designed by Messrs. Kitson and Parish, Mr. E. T. Hall having acted as consulting architect.

The total cost was about £49,000 exclusive of plant and furniture.

## Figures and Tables

**Figure f1:**
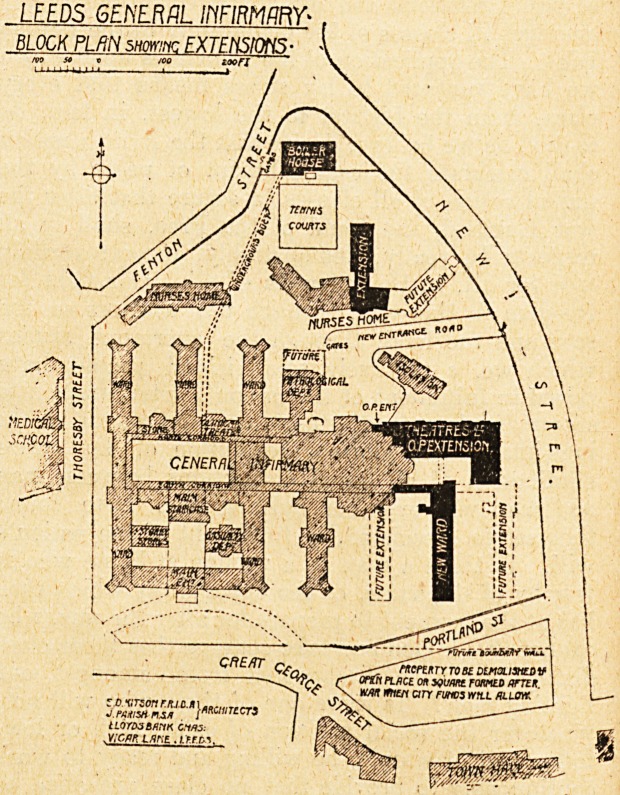


**Figure f2:**
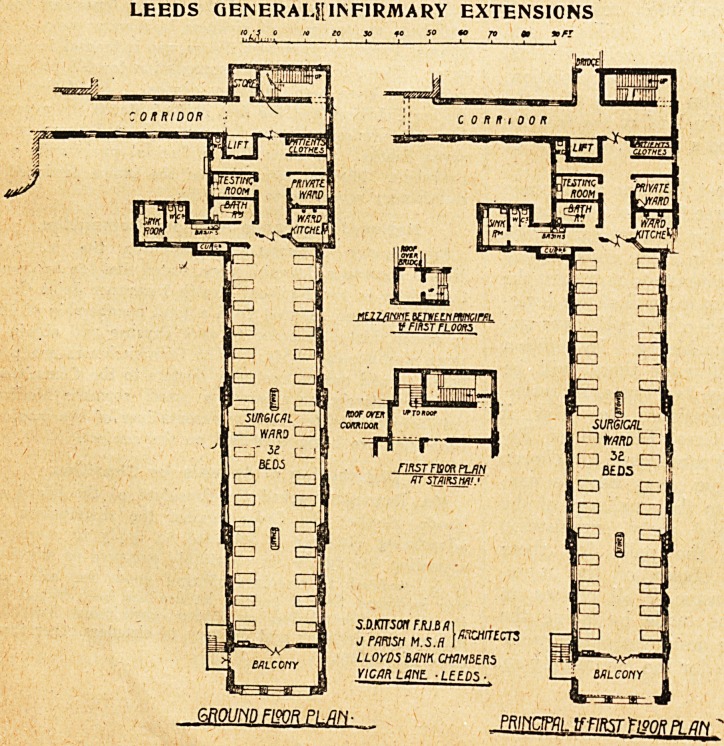


**Figure f3:**
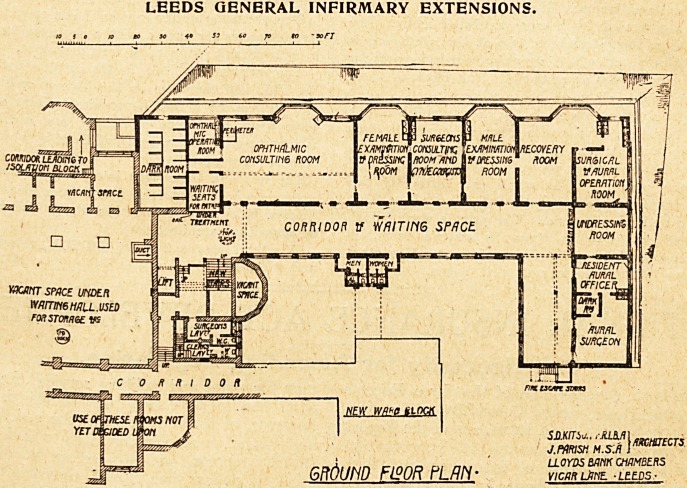


**Figure f4:**